# Investigating Factors Influencing Nurses’ Behavioral Intention to Use Mobile Learning: Using a Modified Unified Theory of Acceptance and Use of Technology Model

**DOI:** 10.3389/fpsyg.2022.673350

**Published:** 2022-05-16

**Authors:** Chen-Ying Su, Cheng-Min Chao

**Affiliations:** ^1^Department of Nursing, National Quemoy University, Jinning, Taiwan; ^2^Department of Business Administration, National Taichung University of Science and Technology, Taichung, Taiwan

**Keywords:** mobile learning, technostress, unified theory of acceptance and use of technology, information quality, system quality, nurse education

## Abstract

The purpose of this study was to develop and empirically test a model for predicting the key factors affecting nurses’ behavioral intention to use mobile learning (m-learning). We explored behavioral intention from users’ perspectives by applying an extended unified theory of acceptance and use of technology (UTAUT) model with the addition of information quality, system quality, technostress, and satisfaction. We conducted a survey of the district and regional hospitals in central Taiwan. Data were derived from 434 respondents. Structural equation modeling was applied to analyze the causal effects of 15 hypothesized predictive factors. We determined that satisfaction, social influence, performance expectancy, facilitating conditions, and effort expectancy positively impacted nurses’ behavioral intention to use m-learning. In addition, technostress was a negative antecedent of effort expectancy. Information quality and system quality had significantly positive effects on satisfaction, performance expectancy, and effort expectancy. This study provides hospital managers with a reference when assessing future developments and informs approaches to promote m-learning.

## Introduction

Following the proliferation of Internet technology and mobile devices, the way people communicate changes. The impact also extends to how people obtain knowledge and information, which leads to how people approach life and work. The revolution in mobile eLearning has taken off. The concept of mobile learning (m-learning) has evolved from distance learning to e-learning (Chao, 2019; Chavoshi and Hamidi, 2019). Using mobile technologies to establish a learning environment that is not limited by time and space, m-learning is a key form of e-learning (Hamidi and Chavoshi, 2018; Chao, 2019). How to best implement m-learning is a vital issue for learners and educational policymakers (Christensen and Knezek, 2018). In recent years, the issues related to m-learning have been widely discussed around the world. M-learning has been defined as “learning that occurs when learners have access to information anytime and anywhere *via* mobile technologies to perform authentic activities in the context of their learning” (Martin and Ertzberger, 2013). However, the development and application of m-learning in nursing education still lack a systematic analysis (Chang et al., 2018; Kim and Park, 2019). Therefore, implementing mobile technology in nursing education may not only helps nursing students learn effectively and improve learning satisfaction and performance within limited clinical learning time but also helps nursing staff conduct training and reinforce professional skills.

Chang et al. (2018) suggested that the application of mobile technology in nursing education can elucidate the behavioral intention of m-learners in nursing education. Wu et al. (2011) developed a mobile guiding system for a clinical nursing course. Through mobile devices, learners were guided to interact with simulated standard patients in real scenarios. This can lower the barrier between practical and theoretical knowledge. The results showed that the nursing students who used the mobile guiding system outperformed those using conventional methods in disease identification, which confirmed that m-learning can effectively improve nursing students’ results. Kim and Park (2019) conducted a systematic review and meta-analysis to assess the effects of smartphone-based m-learning on nurses and nursing students. They found that smartphone-based m-learning may be an alternative method for more effective nursing education. However, most studies were conducted with nursing students rather than with nurses. In sum, understanding nurses’ experiences with m-learning in nursing education and their behavioral intention for its use is valuable.

Recently, mobile technology is widely used in the healthcare industry, include: mobile health education, mobile health (Chang et al., 2018; Duarte and Pinho, 2019; Yu et al., 2021). To understand users’ behaviors concerning technology acceptance, most studies utilized the theory of planned behavior (TPB; Ajzen, 1991), the technology acceptance model (TAM; Davis, 1989), the information system success model (ISSM), and the unified theory of acceptance and use of technology (UTAUT; Venkatesh et al., 2003; Karimi, 2016). In the past, TAM was one of the most prevailing models used to understand technology acceptance. However, under mobile technology learning circumstances, the idea of integrating mobile devices with learning ensures that the learner will consider the social influence and the facilitating conditions owing to the characteristics of m-learning (Wang and Shih, 2009; Briz-Ponce et al., 2017). In short, we utilized the UTAUT model in this research proposed by Venkatesh et al. (2003). The importance of UTAUT lies within it being recognized as the most comprehensive theoretical model with strong explanatory power. UTAUT explains 40 and 70% of the users’ intention of adopting technological solutions (Pappas et al., 2019; Chopik and Francis, 2022). The model also integrates elements across eight prior models, including the TPB (Ajzen, 1991), the TAM (Davis, 1989), the theory of reasoned action (TRA; Fishbein and Ajzen, 1975), a combined version of TPB and TAM (c-TAM-TPB; Taylor and Todd, 1995), a motivational model (MM; Davis et al., 1989), a model of PC utilization (MPCU; Thompson et al., 1991), the innovation diffusion theory (IDT; Rogers, 2003), and the Social cognitive theory (SCT; Bandura, 1986). The UTAUT model integrates performance expectancy, effort expectancy, social influence, and facilitating conditions that directly impact behavioral intention. Therefore, we utilized the UTAUT model in this research.

The ISSM is used to investigate end-user’s computing satisfaction. It includes information quality, system quality, satisfaction, and system use (DeLone and McLean, 1992, 2003). However, previous studies have rarely addressed the impact of information quality and system quality on performance expectancy and effort expectancy, respectively. System use can be noted as the degree and manner in which nurse learners utilize the capabilities of an m-learning system. Therefore, this study integrates two constructs—performance expectancy and effort expectancy—from the UTAUT model to represent system use. By integrating the UTAUT model and the ISSM, this study expects to predict nurses’ behavioral intention to use m-learning in Taiwan. In addition, m-learning is an information communication technology; it has created the stress called “technostress” when nurses use m-learning (Lee et al., 2016). Technostress combines the positive and negative effects of technology tasks to create uncertainty in m-learning: this stress involves both physical and psychological symptoms. This study examined technostress to clarify how it impacts performance and effort expectancy.

Scant research has attempted to identify and analyze external factors and their impact on the main determinants of users’ behavioral intention, such as effort expectancy and performance expectancy. Moreover, factors, such as technostress, have seldom been discussed. This study is thus the first attempt to understand the antecedents of UTAUT in the context of m-learning. The base theoretical models can be extended with different types of external factors, for which different classification schemas have been proposed in the existing literature. Thus, this study extends the constructs to include the ISSM and technostress to further examine the acceptance of m-learning. Specifically, in this study, external factors were classified into (1) system technology factors (such as information quality and system quality) and (2) individual factors (such as nurses’ technostress and satisfaction). Our purpose areas follows: (1) to investigate the factors affecting nurses’ behavioral intention to use m-learning; (2) to the individual differences in the integrating model and to examine the relationships among the ISSM, the UTAUT, and technostress; and (3) to add new external variables to the UTAUT construct, the ISSM (information quality, system quality, and system satisfaction), and technostress, as well as to empirically assess the relationship between this construct and the UTAUT of m-learning.

## Literature Review and Hypotheses

### E-Learning and M-Learning

With rapid Internet infrastructure development, information resources are abundant with users’ access to necessary devices and Internet service. E-learning can be defined as rendering knowledge anytime and anywhere with access to the Internet. Cidral et al. (2018) suggest that “e-learning provides people with a flexible and personalized way to learn; allowing learning on demand and reducing the cost of learning. A variety of core technologies that can facilitate the design and implementation of e-learning systems are emerging, and therefore a far-reaching impact on learning is achieved in the new millennium.”

Along with mobile technology development, e-learning branches out to m-learning. M-learning goes beyond the limitations of e-learning, including that educators and students can use hand-held devices to achieve and perform learning goals—anywhere and at any time—beyond traditional classroom techniques. M-learning also provides an opportunity to render customized learning experiences through the use of online storage applications or online content. For example, e-books, demonstration videos, massive graphics, or article content are available *via* Google suite for Education, which highlights the significant potential for m-learning. It is also worth noting that mobile users have passed desktop users in population and minutes of use; consistently, Chaffey (2018) illustrates that the digital future is on mobile devices. Thus, it is relevant that m-learning is playing a pivotal role in e-learning moving forward.

### Unified Theory of Acceptance and Use of Technology

Venkatesh and Davis (2000) and Venkatesh et al. (2003) developed UTAUT. The UTAUT model is a modified model of the TAM (Venkatesh et al., 2003). TAM suggests that perceived usefulness and perceived ease of use together are thus associated with individuals’ behavioral intention to use technology, which, in turn, leads to actual use (Ajzen and Fishbein, 1980). Therefore, UTAUT adopts the two variables of the TAM and expands them into four constructs: performance expectancy, effort expectancy, social influence, and facilitating conditions. These constructs influence behavioral intention to use technology. Venkatesh et al. (2012) suggested that UTAUT is a “comprehensive synthesis of prior technology acceptance research.” Evolving from TAM, UTAUT also integrates TRA, MM, TPB, C-TAM-TPB, MPCU, IDT, and SCT. UTAUT explains (Chao, 2019; Duarte and Pinho, 2019; Arfi et al., 2021; Chopik and Francis, 2022), more precisely, how people adopt technology and use technology compared to the other related models. “In longitudinal field studies of employee technology acceptance, UTAUT explained about 70 percent of the variance in behavioral intention to use a technology and about 50 percent of the variance in technology use” (Venkatesh et al., 2012).

#### Performance Expectancy

Performance expectancy is the level of expectancy the information technology provides to enhance performance (Arfi et al., 2021; Chopik and Francis, 2022). In m-learning courses for nurses, if the tool can provide a better learning result, or the m-learning experience can escalate work performance, then nurses will have more intention to use it. This is comparable to perceived usefulness in TAM, in which the technology is more accepted when users perceive it as beneficial.

#### Effort Expectancy

Effort expectancy is related to the easiness to use. If it requires less effort to utilize the technology, the more a user will be inclined to use it. According to Magsamen-Conrad et al. (2015), “Effort expectancy refers to the level of ease related to the utilization of the system. Its root constructs are perceived ease of use.” Many researchers have found that effort expectancy has a significant influence on the intention to adopt new technology (Alraja, 2015; Chao, 2019; Arfi et al., 2021). For nurses completing m-learning, how the interface fares with their expectations may lead to their increased or decreased interest.

#### Social Influence

People may follow others’ steps to adopt new technology. The reasons vary, such as to meet expectations, the competition, or being motivated by rewards; however, Alraja (2016) defines social influence as “the degree to which that others (family, friends, peers, etc.) believe (either positive or negative) will affect someone to use the new system.” From Bozan et al.’s (2016) perspective, in the UTAUT model, performance expectancy and effort expectancy are defined as individual factors, and “the social factors—social influence—are more uniform aspects that affect the behavioral intention.” For nurses taking m-learning courses, the primary reasons for completing said courses may be for peer acceptance, to elevate one’s social status, or being forced by the circumstances.

#### Facilitating Conditions

Alraja (2016) argues that “facilitating conditions refers to which extent people believe that an organizational and technical infrastructure exists to support the system.” Thomas et al. (2013) argue that “facilitating conditions significantly affect behavioral intention even when the effects of performance expectancy and effort expectancy on behavioral intention are included.” In m-learning settings, technical support teams can be considered as a facilitating condition that helps users feel comfortable when participating, thus increasing their behavioral intention. In sum, we proposed the following seven hypotheses (H):

H1: An increase in performance expectancy will increase nurses’ behavioral intention to use m-learning.

H2: An increase in effort expectancy will increase nurses’ behavioral intention to use m-learning.

H3: An increase in social influence will increase nurses’ behavioral intention to use m-learning.

H4: An increase in facilitating conditions will increase nurses’ behavioral intention to use m-learning.

H5: An increase in satisfaction will increase nurses’ behavioral intention to use m-learning.

H6: An increase in effort expectancy will increase nurses’ performance expectancy.

H7: An increase in social influence will increase nurses’ performance expectancy.

### Information System Success Model

Information system success model renders a precise identification, description, and explanation of the most critical elements to review system information, which is one of the pivotal scholarly works impacting the Internet era to date. For an information system, the key to success is to improve users’ perception of its quality. DeLone and McLean (1992) established ISSM. It was extended by Seddon (1997), who suggested that system quality and information quality affect perceived usefulness and user satisfaction.

For m-learning to be successful in clinical nursing courses, system quality must reflect m-learning system characteristics. Seddon (1997) defined system quality as being “concerned with whether or not there are bugs in the system, the consistency of the user interface, ease of use, quality of documentation, and sometimes, quality” as well as the “maintainability of the program code.” Stefanovic et al. (2016) argue that system quality should be user-friendly and easy to use. In practical application, the professionalism and complexity of the information will directly affect the user experience. Without a reliable and friendly user interface, participating nurses cannot properly use the m-learning course to obtain necessary information and knowledge, nevertheless fostering nurses’ course satisfaction.

Information quality is correlated with the content of m-learning nursing courses. This includes up-to-date information and accurate and relevant data for participants. Information quality is “concerned with such issues as the relevance, timeliness, and accuracy of the information generated by an information system” (Seddon, 1997). Stefanovic et al. (2016) define information quality as providing precise, up-to-date, sufficient, reliable, and useful information. This will affect participants’ perceived usefulness of the m-learning course, and whether they will be satisfied with the course. Seddon (1997) suggests that user satisfaction is “a subjective evaluation of the various consequences evaluated on a pleasant-unpleasant continuum.” Further, the more satisfied one is with the system itself, the more likely one is to find the system to be easy to use. This notion of “easy to use” thus reinforces effort and performance expectancy. Accordingly, we maintained that information quality and system quality would be correlated with satisfaction, effort expectancy, and performance expectancy. Specifically, we proposed the following six hypotheses:

H8: An increase in information quality will increase nurses’ performance expectancy.

H9: An increase in information quality will increase nurses’ effort expectancy.

H10: An increase in information quality will increase nurses’ satisfaction.

H11: An increase in system quality will increase nurses’ performance expectancy.

H12: An increase in system quality will increase nurses’ effort expectancy.

H13: An increase in system quality will increase nurses’ satisfaction.

### Technostress

Psychiatrists believe that technology triggers human reactions to become a stress. The idea of technostress was first introduced to the field of technology and psychology by Brod (1984): “Technostress is a modern disease of adaptation caused by an inability to cope with the new computer technologies in a healthy manner. It manifests itself in two distinct and related ways: in the struggle to accept computer technology, and in the more specialized form of over-identification with computer technology.” Tarafdar et al. (2007) found that users “feel inundated with information and are forced to work faster to cope with increased processing requirements. Also, they feel compelled to acquire and process the information simply because it is available. This may impair performance and lead to stress.” For m-learning users, courses affect their working hours and their performance will be under scrutiny; thus, we proposed the following hypotheses:

H14: An increase in technostress will decrease nurses’ performance expectancy.

H15: An increase in technostress will decrease nurses’ effort expectancy.

**FIGURE 1:**
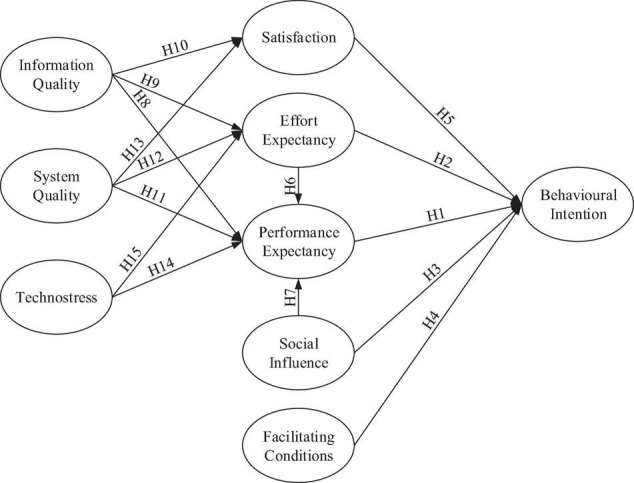
Conceptualized extended UTAUT model for measuring nurses’ use of m-learning.

Figure 1 provides a pictorial depiction of the research framework, which combines two models—UTAUT and ISSM—to elucidate the factors that influence nurses’ behavioral intention to use m-learning. The independent variables were information quality, system quality, technostress, social influence, and facilitating conditions. The dependent variables were satisfaction, effort expectancy, performance expectancy, and behavioral intention. The final construct we measured was behavioral intention to use m-learning, which we posited would be directly influenced by satisfaction, effort expectancy, performance expectancy, social influence, and facilitating conditions—with information quality and system quality additionally influencing users’ level of satisfaction, effort expectancy, and performance expectancy. In addition, technostress would influence effort and performance expectancy. These nine predictors form an extended UTAUT model for predicting behavioral intention.

## Research Methodology

### Instrumentation and Data Collection

This study adopted the back-translation method to ensure the validity of the translation of the questionnaire (Brislin, 1970). We translated the previously developed questionnaire items from English to Chinese since the survey was conducted in Taiwan. We also modified the wording to match the context and the target audience. To reduce any differences in the questionnaire project, items were then back-translated by an experienced m-learning researcher. Then, we performed a pilot test to determine the clarity of the measurement items and improve the face validity of the instrument.

Data were collected by a cross-sectional questionnaire with close-ended questions. The questionnaire was designed in two parts. The first part contained five questions that collected basic information about participants, including age, education level, time of mobile device used, type of hospital, and position. The second part included 47 items to measure the nine variables of the model in the context of m-learning: technostress, information quality, system quality, satisfaction, performance expectancy, effort expectancy, social influence, facilitating conditions, and behavioral intention. All items were adopted from previous literature and modified slightly to fit the current research context. Each item was responded to using a five-point Likert scale—ranging from 1 (strongly disagree) to 5 (strongly agree)—and higher scores indicated higher agreeance with each item.

To develop the instrument for our research purposes, we adapted scales from previous literature. The measuring items for the UTAUT model constructs were adapted from the measurements developed by several previous researchers (Venkatesh et al., 2003, 2012; Alraja, 2015, 2016; Magsamen-Conrad et al., 2015; Karimi, 2016; Chao, 2019; Duarte and Pinho, 2019; Arfi et al., 2021), including performance expectancy (five items), effort expectancy (six items), social influence (six items), facilitating conditions (five items), and behavioral intention (six items). Items for the ISSM variables, that is, information quality, system quality, and satisfaction, were adopted from several previous researchers (DeLone and McLean, 1992, 2003; Seddon, 1997; Stefanovic et al., 2016). The information quality measure contained five items, system quality had five items, and satisfaction had four items. Finally, technostress was measured using five items (Tarafdar et al., 2007; Lee et al., 2016). The measurement items included in each construct are depicted in the Supplementary Appendix Table 1.

### Pilot Test

In the pilot test, we collected data from 96 nurses (N1, N2, N3, and N4) from four hospitals in central Taiwan. Nurses who participated in the pilot test were excluded from the subsequent study. In addition, the pilot test of the questionnaire was conducted using convenience sampling. The results of the pilot study showed that Cronbach’s alphas (α) ranged from 0.911 to 0.955. According to Hair et al. (2010), the Cronbach’s α of each construct should be ≥0.7, indicating the reliability of the questionnaire items used in this study.

### Participants

We recruited 500 nurses from five hospitals (including regional and district hospitals) in Taiwan. These hospitals were classified by the Taiwan Joint Commission on Hospital Accreditation. One hundred nurses were randomly selected from each sample hospital. The lists of nurses provided by the human resources or nursing departments were cleared before sampling by removing nurses on long-term leave (sick leave, maternity leave, study leave, etc.). All participating nurses had experience using m-learning. The data were anonymous and there is no way for readers to identify the participants. In addition, all participants were volunteers and could refuse participation at any time without consequences. In total, 472 completed questionnaires were submitted.

The research team reviewed the questionnaires and discarded the questionnaires that were incomplete/missing data. Finally, 434 useable questionnaires were obtained (valid response rate = 86.8%, 434/500). Among the valid responses, 265 (61.1%) were from district hospitals and 169 (38.9%) were from regional hospitals. Nurses’ mean age was 31.06 years (SD = 5.65), with a range from 22 to 50 years. Three-quarters had a faculty degree/bachelor’s degree (*n* = 325), 13.4% (*n* = 58) had a nursing college degree, and 11.8% (*n* = 51) had a master’s degree or above. Most participants (89.2%) declared that they had used a mobile device for more than 3 years, followed by 2 to 3 years (6.2%). About half (46.1%) had an N2 (*n* = 200), followed by an N1 (35.9%, *n* = 156; see Table 1).

**TABLE 1:** Profiles of respondents (*N* = 434).

Factor/level	*N*	%	Factor/level	*N*	%
*Formal education*			*Type of hospital*		
Nursing college	58	13.4	District hospitals	265	61.1
Faculty degree/bachelor degree	325	74.9	Regional hospitals	169	38.9
Master degree or above	51	11.8	*Position*		
*Time of mobile device used*			N1	156	35.9
Less than 1 years	10	2.3	N2	200	46.1
1–2 (included) years	10	2.3	N3	39	9.0
2–3 (included) years	27	6.2	N4	39	9.0
More than 3 years	387	89.2			

## Results

### Data Analysis

We used a structural equation model (SEM) to conduct this research, and we employed partial least squares (PLS) software to analyze the proposed conceptual model and test the hypotheses. In addition, this study followed the two-stage analytical technique suggested by Hair et al. (2017), which are: (1) the measurement model assessment (validity and reliability) and (2) the structural model assessment (testing the hypothesized relationships).

### Measurement Model Evaluation

This study first examined the measurement model to test the internal reliability, convergent validity, and discriminant validity (DV). To evaluate the internal reliability for all constructs, Cronbach’s α and composite reliability (CR) were used. To assess the convergent validity for all constructs, average variance extracted (AVE) was used (Fornell and Larcker, 1981; Hair et al., 2010; Bagozzi and Yi, 2012).

As shown in Table 2, the factor loadings of all items were higher than the recommended levels of 0.7, and they were all significant (*p* < 0.05; Hair et al., 2010). Cronbach’s α ranged from 0.916 to 0.950, thereby suggesting high internal reliability. The CR values ranged from 0.937 to 0.962, exceeding the recommended cutoff of 0.7 (Fornell and Larcker, 1981; Hair et al., 2010). The AVE values ranged from 0.719 to 0.834, exceeding 0.5 for each construct (Fornell and Larcker, 1981). Finally, concerning DV, the values of all constructs were greater than 1.0, indicating an appropriate level of DV (Hair et al., 2017).

**TABLE 2:** Construct reliability results.

Construct	No. of items	Item loading	Cronbach’s α	AVE	CR	DV
Technostress	5	0.830∼0.949	0.950	0.834	0.962	10.487
Information quality	5	0.801∼0.902	0.916	0.748	0.937	1.668
System quality	5	0.852∼0.912	0.925	0.769	0.943	1.714
Satisfaction	4	0.862∼0.935	0.926	0.819	0.948	1.379
Performance expectancy	5	0.775∼0.916	0.916	0.752	0.938	1.266
Effort expectancy	6	0.866∼0.931	0.944	0.817	0.957	2.173
Social influence	6	0.792∼0.938	0.937	0.763	0.951	1.614
Facilitating conditions	5	0.830∼0.919	0.928	0.778	0.946	1.822
Behavioral intention	6	0.772∼0.885	0.921	0.719	0.939	1.426

*AVE, average variance extracted; CR, composite reliability; DV, discriminant validity.*

### Statistical Analysis and Hypotheses Testing

Regarding the overall quality of the research model, the SEM procedure based on PLS regression was applied to analyze the goodness of fit (GoF), path coefficients, and coefficient of determination (*R*^2^). Alolah et al. (2014) suggested using GoF as a global fit metric for PLS path modeling. Tenenhaus et al. (2005) suggested that GoF must be higher than the proposed 0.36 benchmark. According to the above results, the GoF value was 0.581, exceeding this noted the cut-off and indicating that the model structure was fitted to the data.

**FIGURE 2:**
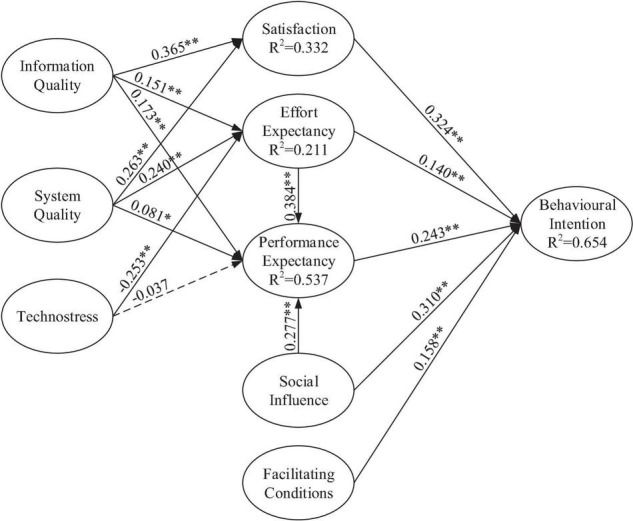
Extended UTAUT with latent variables and path coefficients. Value on path: standardized coefficients (β), *R*^2^: Coefficient of determination and ^∗^*p* < 0.05, ^∗∗^*p* < 0.01.

The model had five exogenous variables (information quality, system quality, technostress, social influence, and facilitating conditions) and four endogenous variables (satisfaction, performance expectancy, effort expectancy, and behavioral intention). Table 3 and Figure 2 present the hypothesis testing results, the amount of variance explained (*R*^2^), the standardized path coefficients for each hypothesized path, and the associated *t*-values for each construct. According to the path analysis, 14 of the 15 hypotheses were supported in the model.

**TABLE 3:** Estimation results for Hypotheses 1 to 15.

Hypotheses	Path from/to	Standardized coefficient	*t*-value	Test results
H 1	Performance expectancy → Behavioral intention	0.243**	4.562	Supported
H 2	Effort expectancy → Behavioral intention	0.140**	3.674	Supported
H 3	Social influence → Behavioral intention	0.310**	6.423	Supported
H 4	Facilitating conditions → Behavioral intention	0.158**	3.592	Supported
H 5	Satisfaction → Behavioral intention	0.324**	5.619	Supported
H 6	Effort expectancy → Performance expectancy	0.384**	9.790	Supported
H 7	Social influence → Performance expectancy	0.277**	5.630	Supported
H 8	Information quality → Performance expectancy	0.173**	3.595	Supported
H 9	Information quality → Effort expectancy	0.151**	2.763	Supported
H 10	Information quality → Satisfaction	0.365**	7.597	Supported
H 11	System quality → Performance expectancy	0.081*	1.995	Supported
H 12	System quality → Effort expectancy	0.240**	5.057	Supported
H 13	System quality → Satisfaction	0.263**	5.382	Supported
H 14	Technostress → Performance expectancy	–0.037	1.078	Non-supported
H 15	Technostress → Effort expectancy	−0.253**	6.324	Supported

*p < 0.05, **p < 0.01.

In the final model, information quality and system quality were all crucial antecedents of satisfaction (βs = 0.365 and 0.263, respectively), thereby supporting H10 and H13. In summary, information quality and system quality variables jointly explained 33.2% of the variance in the satisfaction variable (*R*^2^ = 0.332). Information quality, system quality, and technostress were significant determinants of effort expectancy (βs = 0.151, 0.240, and −0.253, respectively), thereby supporting H9, H12, and H15. These three constructs explained 21.1% of the variance in the effort expectancy variable (*R*^2^ = 0.211). Effort expectancy, social influence, information quality, and system quality were significant determinants of performance expectancy (βs = 0.384, 0.277, 0.173, and 0.081, respectively), thereby supporting H6 to H8 and H11. However, technostress had a non-significant effect on performance expectancy, thereby not supporting H14. Therefore, four constructs explained 53.7% of the variance in the performance expectancy variable (*R*^2^ = 0.537): effort expectancy, social influence, information quality, and system quality. Finally, performance expectancy, effort expectancy, social influence, facilitating conditions, and satisfaction were significantly positive determinants of behavioral intention (βs = 0.243, 0.140, 0.310, 0.158, and 0.324, respectively), thereby supporting H1 to H5. These five constructs explained 65.4% of the variance in the behavioral intention variable (*R*^2^ = 0.654). In sum, satisfaction had the most significant effect on behavioral intention, followed by social influence, performance expectancy, and effort expectancy, respectively.

## Discussion

The conceptual model of this study combined two models—UTAUT and ISSM—to clarify the factors that affect nurses’ behavioral intention to use m-learning in Taiwanese hospitals. Indeed, this study successfully integrated factors from both models into this research framework. This study found that the four UTAUT constructs—effort expectancy, performance expectancy, social influence, and facilitating conditions—and one of the ISSM constructs—satisfaction—influenced nurses’ behavioral intention to use m-learning. Further, two of the ISSM constructs—information quality and system quality—influenced nurses’ satisfaction, effort expectancy, and performance expectancy. Technostress also influenced nurses’ effort expectancy. These results highly support the predictive validity of the current model.

According to the path coefficient analyses, the four factors from the UTAUT had significant relationships with behavioral intention. This finding is consistent with earlier research (Venkatesh et al., 2003, 2012; Alraja, 2015; Magsamen-Conrad et al., 2015; Chao, 2019; Duarte and Pinho, 2019; Arfi et al., 2021; Chopik and Francis, 2022). The social influence had the most significant effect and was among the strongest predictors of behavioral intention to use m-learning, because its adoption depends very much on nurses’ level of engagement, hospital, and the government health-related department. Nurses must receive a certain amount of education and training each year; therefore, hospitals should provide a convenient m-learning environment and support the use of m-learning. If m-learning is mandatory and nurses express the need for such systems, most will likely use m-learning.

The results also showed that performance expectancy was a significant predictor of behavioral intention to use m-learning. Most nurses do not have enough time to acquire learning, training, and reinforce their professional skills; therefore, they may be looking at m-learning as an effective solution. In addition, facilitating conditions significantly affected nurses’ behavioral intention to use m-learning, which could reflect the extent to which nurses use mobile technology and information and communication technologies resources in m-learning. Consequently, they are aware of the importance of mobile technical and infrastructural resource requirements and their impact on behavioral intention to use m-learning.

Previous studies (Davis et al., 1989; Venkatesh and Davis, 1996; Liu et al., 2010) suggest that, to better explain the adoption of technology, it is important to understand the antecedents of the key constructs. However, little is known about the impact of information quality and system quality on performance expectancy and effort expectancy, respectively. Therefore, this study used ISSM, satisfaction, information quality, and system quality as antecedents of the key constructs of UTAUT. We found that satisfaction was a key determinant on nurses’ behavioral intention to use m-learning. Moreover, information quality and system quality had a positive impact on satisfaction, corroborating similar results on the success of e-learning systems (DeLone and McLean, 1992, 2003; Stefanovic et al., 2016). Information quality and system quality also had positive impacts on effort expectancy and performance expectancy. During the m-learning process, if the m-learning platform is easy to use while providing nurses useful, updated, and correct information applicable at work. Therefore, nurses will consider m-learning a valuable tool to improve their job performance and easy to use. Said feelings toward the system increased nurses’ system satisfaction, effort expectancy, and performance expectancy. Subsequently, they felt that m-learning helped them learn and enhance their learning results, which elevated their behavioral intention to use m-learning. In addition, nurses thought more favorably of m-learning system quality if they perceived the m-learning system as offering complete functions, was easy to understand and manipulate, and was stable. Said feelings elevated their m-learning satisfaction, effort expectancy, and performance expectancy, thereby elevating their behavioral intention to use m-learning.

The role of technostress had not been previously analyzed in the context of behavioral intention to use m-learning. In this study, technostress negatively influenced effort expectancy; however, it had no significant impact on performance expectancy. The results imply that nurses who are stressed because they feel that they are unable to keep up with new information technology are reluctant to utilize m-learning, which lowers their effort expectancy. They may even consider m-learning to have no positive effects on their learning. Thus, hospitals are recommended to lower nurses’ technostress before promoting m-learning and incorporating new technology when providing educational training.

## Conclusion

This study was to develop and empirically extend the UTAUT model with the addition of information quality, system quality, technostress, and satisfaction to offer a more comprehensive explanation for the key factors affecting nurses’ behavioral intention to use m-learning. Therefore, the purpose of this study are as follows: (1) to investigate the factors affecting nurses’ behavioral intention to use m-learning; (2) to the individual differences in the integrating model and to examine the relationships among ISSM, UTAUT, and technostress; and (3) to add new external variables to the UTAUT construct, the ISSM (information quality, system quality, and system satisfaction), and technostress, as well as to empirically assess the relationship between this construct and the UTAUT of m-learning. To this end, data collected from a sample of 434 respondents in regional hospitals in central Taiwan were analyzed using structural equation modeling. The results revealed that satisfaction, social influence, performance expectancy, facilitating conditions, and effort expectancy are important antecedent factors in nurses’ behavioral intention to use m-learning. In addition, technostress was a negative antecedent of effort expectancy. Finally, information quality and system quality had significantly positive effects on satisfaction, performance expectancy, and effort expectancy. This study provides hospital managers with a reference when assessing future developments and informs approaches to promote m-learning.

## Limitations and Suggestions for Further Studies

This study had some limitations that future research should address. First, data were collected using a cross-sectional design. Therefore, future studies should consider using a longitudinal design, which may result in more accurate findings from specific groups. In addition, different methods can be used as Quantum Machine Learning Architecture (Amin et al., 2021). Although we considered several theories to devise our model, we did not include expectations-confirmation theory, flow theory, UTAUT 2, mobile literacy, or mobile self-efficacy. Future research may wish to do so to produce a more comprehensive study. Finally, this study was conducted at five hospitals in Taiwan; thus, the model may not be generalizable to other areas. Future research should expand data to investigate long-term effects. In addition, the model should be tested in other countries and refined accordingly to improve its predictive power.

## Data Availability Statement

The original contributions presented in the study are included in the article/Supplementary Material, further inquiries can be directed to the corresponding author.

## Author Contributions

C-YS collected the data, carried out the data curation and statistical analysis, obtained the funding, and wrote the original draft. C-MC did the research conceptualization, collected the data, carried out the statistical analysis, interpreted the data, supervised the study, obtained the funding, and wrote the original draft. Both authors wrote the manuscript together and approved the final manuscript.

## Conflict of Interest

The authors declare that the research was conducted in the absence of any commercial or financial relationships that could be construed as a potential conflict of interest.

## Publisher’s Note

All claims expressed in this article are solely those of the authors and do not necessarily represent those of their affiliated organizations, or those of the publisher, the editors and the reviewers. Any product that may be evaluated in this article, or claim that may be made by its manufacturer, is not guaranteed or endorsed by the publisher.

## Supplementary Material

Supplementary Appendix Table 1 The research questionnaire.

## References

[B1] Ajzen I. (1991). The theory of planned behavior. *Organ. Behav. Hum. Decis. Process.* 50 179–211.

[B2] Ajzen I. Fishbein M. (1980). *Understanding Attitudes And Predicting Social Behaviour.* Englewood Cliffs, NJ: Prentice Hall.

[B3] Alolah T. Stewart R. A. Panuwatwanich K. Mohamed S. (2014). Determining the causal relationships among balanced scorecard perspectives on school safety performance: case of Saudi Arabia. *Accid. Anal. Prevent.* 68 57–74. 10.1016/j.aap.2014.02.002 24589246

[B4] Alraja M. N. (2015). User acceptance of information technology: a field study of an e-mail system adoption from the individual students’. *Perspective. Mediterr. J. Soc. Sci.* 6:19.

[B5] Alraja M. N. (2016). The effect of social influence and facilitating conditions on e-government acceptance from the individual employees’ perspective. *Pol. J. Manag. Stud.* 14 18–27. 10.17512/pjms.2016.14.2.02

[B6] Amin J. Sharif M. Gul N. Kadry S. Chakraborty C. (2021). Quantum machine learning architecture for COVID-19 classification based on synthetic data generation using conditional adversarial neural network. *Cogn. Comput.* 10, 1–12. 10.1007/s12559-021-09926-6 34394762 PMC8353617

[B7] Arfi W. B. Nasr I. B. Kondrateva G. Hikkerova L. (2021). The role of trust in intention to use the IoT in eHealth: application of the modified UTAUT in a consumer context. *Technol. Forecast. Soc. Change* 167:120688. 10.1016/j.techfore.2021.120688

[B8] Bagozzi R. P. Yi Y. (2012). Specification, evaluation, and interpretation of structural equation models. *J. Acad. Market. Sci.* 40 8–34. 10.1007/s11747-011-0278-x

[B9] Bandura A. (1986). Fearful expectations and avoidant actions as coeffects of perceived self-inefficacy. *Am. Psychol.* 41 1389–1391. 10.1037/0003-066x.41.12.1389

[B10] Bozan K. Parker K. Davey B. (2016). “A closer look at the social influence construct in the UTAUT model: An institutional theory based approach to investigate health IT adoption patterns of the elderly,” in *Paper Presented At The System Sciences (HICSS), 2016 49th Hawaii International Conference*, Koloa, HI, USA.

[B11] Brislin R. W. (1970). Back-translation for cross-cultural research. *J. Cross Cult. Psychol.* 1 185–216. 10.1037/a0021453 21038953

[B12] Briz-Ponce L. Pereira A. Carvalho L. Juanes-Méndez J. A. García-Peñalvo F. J. (2017). Learning with mobile technologies–Students’ behavior. *Comput. Hum. Behav.* 72 612–620. 10.1016/j.chb.2016.05.027

[B13] Brod C. (1984). *Technostress: The Human Cost Of The Computer Revolution.* Reading, MA: Addison Wesley.

[B14] Chaffey D. (2018). *Mobile Marketing Statistics 2018.* Available online at: https://www.smartinsights.com/mobile-marketing/mobile-marketinganalytics/mobilemarketing-statistics (accessed January 18, 2018).

[B15] Chang C. Y. Lai C. L. Hwang G. J. (2018). Trends and research issues of mobile learning studies in nursing education: a review of academic publications from 1971 to 2016. *Comput. Educ.* 116 28–48. 10.1016/j.compedu.2017.09.001

[B16] Chao C. M. (2019). Factors determining the behavioral intention to use mobile learning: an application and extension of the UTAUT model. *Front. Psychol.* 10:1652. 10.3389/fpsyg.2019.01652 31379679 PMC6646805

[B17] Chavoshi A. Hamidi H. (2019). Social, individual, technological and pedagogical factors influencing mobile learning acceptance in higher education: a case from Iran. *Telemat. Inform.* 38 133–165. 10.1016/j.tele.2018.09.007

[B18] Chopik W. J. Francis J. (2022). Partner influences on ICT use variety among middle-aged and older adults: the role of need for cognition. *Comput. Hum. Behav.* 126:107028. 10.1016/j.chb.2021.107028 34658501 PMC8516131

[B19] Christensen R. Knezek G. (2018). Reprint of readiness for integrating mobile learning in the classroom: challenges, preferences and possibilities. *Comput. Hum. Behav.* 78 379–388. 10.1016/j.chb.2017.07.046

[B20] Cidral W. A. Oliveira T. Di Felice M. Aparicio M. (2018). E-learning success determinants: brazilian empirical study. *Comput. Educ.* 122 273–290.

[B21] Davis F. D. (1989). Perceived usefulness, perceived ease of use, and user acceptance of information technology. *MIS Q.* 13 319–340. 10.2307/249008

[B22] Davis F. D. Bagozzi R. P. Warshaw P. R. (1989). User acceptance of computer technology: a comparison of two theoretical models. *Manag. Sci.* 35 982–1003. 10.1287/mnsc.35.8.982 19642375

[B23] DeLone W. H. McLean E. R. (1992). Information systems success: the quest for the dependent variable. *Inform. Syst. Res.* 3 60–95. 10.1287/isre.3.1.60 19642375

[B24] DeLone W. H. McLean E. R. (2003). The DeLone and McLean model of information systems success: a ten-year update. *J. Manag. Inform. Syst.* 19 9–30.

[B25] Duarte P. Pinho J. C. (2019). A mixed methods UTAUT2-based approach to assess mobile health adoption. *J. Bus. Res.* 102 140–150. 10.1016/j.jbusres.2019.05.022

[B26] Fishbein M. Ajzen I. (1975). *Belief, Attitude, Intention And Behavior: An Introduction To Theory And Research.* Reading, MA: Addison-Wesley.

[B27] Fornell C. Larcker D. F. (1981). Evaluating structural equation models with unobservable variables and measurement error. *J. Market. Res.* 18 39–50. 10.2307/3151312

[B28] Hair J. F. Black W. C. Babin B. J. Anderson R. E. (2010). *Multivariate Data Analysis: A Global Perspective*, 7th Edn. New York, NY: MacMillan.

[B29] Hair J. F. Hult G. T. M. Ringle C. Sarstedt M. (2017). *A Primer on Partial Least Squares Structural Equation Modeling (PLS-SEM)*, 2 Edn. London: SAGE Publications.

[B30] Hamidi H. Chavoshi A. (2018). Analysis of the essential factors for the adoption of mobile learning in higher education: a case study of students of the University of Technology. *Telemat. Inform.* 35 1053–1070. 10.1016/j.tele.2017.09.016

[B31] Karimi S. (2016). Do learners’ characteristics matter? An exploration of mobile-learning adoption in self-directed learning. . *Comput. Hum. Behav.* 63 769–776. 10.1016/j.chb.2016.06.014

[B32] Kim J. H. Park H. (2019). Effects of smartphone-based mobile learning in nursing education: a systematic review and meta-analysis. *Asian Nurs. Res.* 13 20–29. 10.1016/j.anr.2019.01.005 30659927

[B33] Lee S. B. Lee S. C. Suh Y. H. (2016). Technostress from mobile communication and its impact on quality of life and productivity. *Total Qual. Manag. Bus. Excell.* 27 775–790.

[B34] Liu I. F. Chen M. C. Sun Y. S. Wible D. Kuo C. H. (2010). Extending the TAM model to explore the factors that affect Intention to use an online learning community. *Comput. Educ.* 54 600–610. 10.1016/j.compedu.2009.09.009

[B35] Magsamen-Conrad K. Upadhyaya S. Joa C. Y. Dowd J. (2015). Bridging the divide: using UTAUT to predict multigenerational tablet adoption practices. *Comput. Hum. Behav.* 50 186–196. 10.1016/j.chb.2015.03.032 25937699 PMC4412023

[B36] Martin F. Ertzberger J. (2013). Here and now mobile learning: an experimental study on the use of mobile technology. *Comput. Educ.* 68 76–85. 10.1016/j.compedu.2013.04.021

[B37] Pappas I. O. Giannakos M. N. Sampson D. G. (2019). Fuzzy set analysis as a means to understand users of 21st-century learning systems: the case of mobile learning and reflections on learning analytics research. *Comput. Hum. Behav.* 92 646–659. 10.1016/j.chb.2017.10.010

[B38] Rogers E. M. (2003). *The Diffusion Of Innovation*, 5th Edn. New York, NY: Free Press.

[B39] Seddon P. B. (1997). A respecification and extension of the DeLone and McLean model of IS success. *Inform. Syst. Res.* 8 240–253. 10.1287/isre.8.3.240 19642375

[B40] Stefanovic D. Marjanovic U. Deliæ M. Culibrk D. Lalic B. (2016). Assessing the success of e-government systems: an employee perspective. *Inform. Manag.* 53 717–726. 10.1016/j.im.2016.02.007

[B41] Tarafdar M. Tu Q. Ragu-Nathan B. S. Ragu-Nathan T. (2007). The impact of technostress on role stress and productivity. *J. Manag. Inform. Syst.* 24 301–328. 10.1016/j.puhe.2020.09.013 33166856

[B42] Taylor S. Todd P. A. (1995). Understanding information technology usage: a test of competing models. *Inform. Syst. Res.* 6 144–176. 10.1287/isre.6.2.144 19642375

[B43] Tenenhaus M. Vinzi V. E. Chatelin Y. M. Lauro C. (2005). PLS path modeling. *Comput. Statist. Data Anal.* 48 159–205.

[B44] Thomas T. Singh L. Gaffar K. (2013). The utility of the UTAUT model in explaining mobile learning adoption in higher education in Guyana. *Int. J. Educ. Dev. ICT* 9 71–85.

[B45] Thompson R. L. Higgins C. A. Howell J. M. (1991). Personal computing: toward a conceptual model of utilization. *MIS Q.* 125–143. 10.2307/249443

[B46] Venkatesh V. Davis F. D. (1996). A model of the antecedents of perceived ease of use: development and test. *Decis. Sci.* 27 451–481.

[B47] Venkatesh V. Davis F. D. (2000). A theoretical extension of the technology acceptance model: four longitudinal field studies. *Manag. Sci.* 46 186–204. 10.1287/mnsc.46.2.186.11926 19642375

[B48] Venkatesh V. Morris M. G. Davis G. B. Davis F. D. (2003). User acceptance of information technology: toward a unified view. *MIS Q.* 425–478. 10.2307/30036540

[B49] Venkatesh V. Thong J. Y. Xu X. (2012). Consumer acceptance and use of information technology: extending the unified theory of acceptance and use of technology. *MIS Q.* 36 157–178. 10.2307/41410412

[B50] Wang Y. S. Shih Y. W. (2009). Why do people use information kiosks? A validation of the Unified theory of acceptance and use of technology. *Gov. Inform. Q.* 26 158–165. 10.1016/j.giq.2008.07.001

[B51] Wu P. H. Hwang G. J. Tsai C. C. Chen Y. C. Huang Y. M. (2011). A pilot study on conducting mobile learning activities for clinical nursing courses based on the repertory grid approach. *Nurs. Educ. Today* 31 e8–e15. 10.1016/j.nedt.2010.12.001 21196068

[B52] Yu C. W. Chao C. M. Chang C. F. Chen R. J. Chen P. C. Liu Y. X. (2021). Exploring behavioral intention to use a mobile health education website: an extension of the UTAUT 2 model. *SAGE Open* 11 1–12. 10.1504/ijmc.2023.10041673 35009967

