# Supplementary material for: Investigating Factors Influencing Nurses’ Behavioral Intention to Use Mobile Learning: Using a Modified Unified Theory of Acceptance and Use of Technology Model
Source: Front Psychol. 2022 May 16;13:673350. doi: 10.3389/fpsyg.2022.673350 (PMC9150498; doi:10.3389/fpsyg.2022.673350)
Supplement: Supplementary Appendix Table 1 — The research questionnaire. [file Presentation_1.pdf]

Appendix A. The research questionnaire

Constructs	Items	Sources
Technostress	I feel drained from tasks that require me to use my mobile phone.	Lee et al. (2016); Tarafdar et al. (2007).
	I feel tired from using my mobile phone activities.	
	Interacting all day with my mobile phone is a strain for me.	
	I feel burned out from using my mobile phone activities.	
	I feel my personal life is being invaded by mobile phone activities.	
Information Quality	The mobile learning system provides up-to-date information.	DeLong & McLean (2003, 1992); Stefanovic et al. (2016).
	The mobile learning system provides information that is easy to understand	
	The mobile learning system provides information that is exactly what you need.	
	The mobile learning system provides information that is relevant to your learning.	
	The mobile learning system provides sufficient information.	
System Quality	The mobile learning system provides high availability.	DeLong & McLean (2003, 1992); Stefanovic et al. (2016).
	The mobile learning system provides interactive features between users and system.	
	The mobile learning system is well structured.	
	The mobile learning system is easy to use.	
	The mobile learning system has attractive features to appeal to the users.	
Satisfaction	I was very content with the mobile learning system.	DeLong & McLean (2003, 1992); Seddon (1997); Stefanovic et al. (2016).
	I was very pleased with the mobile learning system.	
	I was satisfied with the efficiency of the mobile learning system.	
	I felt delighted with mobile learning system.	
	Overall, I was satisfied with the mobile learning system.	
Performance Expectancy	Using the mobile learning system would improve my learning performance.	Arfi et al. (2021); Chao (2019); Duarte, & Pinho (2019); Karimi (2016); Magsamen-Conrad et al. (2015); Venkatesh et al.
	Using the mobile learning system increased my chances of achieving learning that was important to me.	
	The mobile learning system is useful for my learning.	

Constructs	Items	Sources
	Using the mobile learning system would allow me to accomplish learning tasks more quickly.	(2012); Venkatesh et al. (2003).
	Using the mobile learning system would enhance my effectiveness in learning.	
Effort Expectancy	Learning how to use mobile learning is easy for me.	
	My interaction with the mobile learning would be clear and understandable.	
	I find mobile learning easy to use.	
	I would find it easy to get the mobile learning to do what I want it to do.	
	It is easy for me to become skillful at using mobile learning.	
	Overall, I believe that it is easy to use the mobile learning system to support my learning.	
Social Influence	People who influence my behavior think that I should use the mobile learning system.	
	People who are important to me think that I should use the mobile learning system.	
	People whose opinions that I value prefer that I use the mobile learning system.	
	My working environment influences my intention to use the mobile learning system.	
	In general, the hospital encourages and supports the use of the mobile learning system in providing learning.	
	In general, the manager would support the use of the mobile learning system.	
Facilitating Conditions	I have the resources necessary to use the mobile learning system.	
	I have the knowledge necessary to use the mobile learning system.	
	The mobile learning system is compatible with other forms of learning technologies I use.	
	I can get help from others when I have difficulties using the mobile learning system.	
	I do not need assistance to use the mobile learning system.	
Behavioral	Assuming I had access to the mobile learning system, I	

Constructs	Items	Sources
Intention	intend to use it.	
	Given that I had access to the mobile learning system, I predict that I would use it.	
	If the mobile learning system becomes available permanently, I plan to use it.	
	I intend to use the mobile learning system whenever possible.	
	I will always try to use the mobile learning system in my academic studies.	
	I plan to use the mobile learning system in the future.	
